# Building National Wastewater Surveillance for Infectious Diseases in the United States

**DOI:** 10.3201/eid3213.260301

**Published:** 2026-08

**Authors:** Jonathan S. Yoder, Nicole Fehrenbach, Alexander Yu, David A. Larsen, Scott Santibanez, Margaret A. Honein

**Affiliations:** Centers for Disease Control and Prevention, Atlanta, Georgia, USA (J.S. Yoder, N. Fehrenbach, S. Santibanez, M.A. Honein); California Department of Public Health, Division of Communicable Disease Control, Richmond, California, USA (A. Yu); Syracuse University Department of Public Health, Syracuse, New York, USA (D.A. Larsen)

**Keywords:** wastewater surveillance, bacteria, viruses, parasites, United States

In 2020, the Centers for Disease Control and Prevention (CDC) established the National Wastewater Surveillance System to detect and quantify SARS-CoV-2 RNA through wastewater in the United States. Subsequently, wastewater surveillance (WS) for infectious diseases expanded dramatically from independent, local efforts into a coordinated national system (*1*). This supplemental issue of *Emerging Infectious Diseases* describes the substantial progress during the first 5 years of the National Wastewater Surveillance System, including the dramatic growth of WS epidemiology and the increasing public health applications of WS for infectious diseases. Innovation is occurring in multiple sectors, including frontline health departments, which is reflected in the supplement article authorship. WS has quickly developed into a valuable tool for responding to infectious disease threats.

WS is unique in that it does not depend on whether persons have symptoms, have access to healthcare, visit a provider, or have a clinical diagnostic test performed. As such, it can fill gaps in other surveillance systems, most of which are reliant on clinical diagnostic tests being performed and reported to public health. WS also can act as an early warning system and detect small changes in community transmission of infectious diseases, helping public health agencies take action to prevent further infections. 

WS is funded by CDC but relies on a multitude of partners, including academic researchers, commercial laboratories, community stakeholders, wastewater operators and engineers, healthcare professionals, and public health professionals, as seen throughout the articles in this supplement. Key to jurisdictional involvement are the Wastewater Monitoring Centers of Excellence, regional leaders who foster innovation, develop and conduct training, and provide technical assistance to jurisdictions. The 6 Centers of Excellence are led by public health departments through a collaborative partnership with academic and utility partners in California, Colorado, Houston (Texas), New York, North Carolina, and Wisconsin (*1*).

This network of partners (public health, academia, and utilities) is necessary to navigate the complex process of operationalizing WS and making it useful for public health. The WS process involves collecting untreated wastewater from a sewershed, often as it flows into a wastewater treatment plant. Wastewater samples are typically collected by wastewater treatment plant operators and sent to laboratories for testing, which includes sample preparation and concentration and extraction and quantification of viral genetic material, most commonly using reverse transcription digital PCR or reverse transcription droplet digital PCR. Isolates can be further sequenced to compare with clinical sequences and elucidate the spread of variants. Several sequence-based detection approaches (e.g., hybrid capture or shotgun metagenomics) are being explored to increase the usefulness of WS.

Laboratories submit data to the health department, commercial contractor, or academic partner program, who then send data to CDC through the One CDC Data Platform, a cloud-based data integration and management platform. Frontline jurisdictions are able to immediately access and use their data for public health action while sharing it securely with CDC. CDC currently receives data from ≈1,500 wastewater sampling sites across the United States in all 50 states, 7 territories, and some tribal communities.

As noted in the articles in this supplement, WS data complement, but do not replace, other public health surveillance data. Health departments use wastewater data for a multitude of reasons, including to confirm trends, provide early warning about transmission, inform public health messages, monitor disease activity in communities with limited clinical testing or reporting, direct prevention efforts (e.g., vaccination), inform resource allocation decisions, provide insight on the presence and scope of emerging and rare infectious diseases, and track the emergence of variants. Hospital systems use the information to plan for upcoming increases in visits and hospitalizations, whereas healthcare providers use it to increase their own awareness of locally circulating diseases and refine their differential diagnoses. Individual persons can use WS data to make decisions about their risk for disease and how best to protect themselves and their community. WS is adaptable, can meet changing public health needs, and can rapidly adjust to address emerging and reemerging health threats, such as measles (*2*). However, that flexibility requires a sustained national WS system; ongoing partnerships; continued investment in innovation for different diseases, approaches (e.g., metagenomics), and settings (e.g., nonsewered communities); ongoing evaluation of population and geographic coverage of the system; improvements in timeliness; and better ways to combine data from multiple disparate laboratory sources.

The authors of the articles in this supplement summarize some of the current perspectives on WS for infectious diseases and implications for public health in the United States. Themes include innovations in WS and epidemiology; comparison of WS data with clinical and syndromic surveillance; evaluation of sampling frequency and cadence; representativeness at county, state, territory, and national levels; case studies of specific programmatic applications in public health surveillance and outbreak response in state and local jurisdictions; and critical gaps and directions for the future, such as the impact and future potential of coordinated WS in shaping public health strategies. As the field of public health evolves to face new threats and challenges, data from WS and other innovative sources will be essential in ensuring we are prepared to address emerging and reemerging infectious diseases in the 21st Century.
